# lncRNA-disease association prediction based on the weight matrix and projection score

**DOI:** 10.1371/journal.pone.0278817

**Published:** 2023-01-03

**Authors:** Bo Wang, Chao Zhang, Xiao-xin Du, Xiao-dong Zheng, Jing-you Li

**Affiliations:** College of Computer and Control Engineering, Qiqihar University, Qiqihar, People’s Republic of China; Indian Institute of Technology Patna, INDIA

## Abstract

With the development of medical science, long noncoding RNA (lncRNA), originally considered as a noise gene, has been found to participate in a variety of biological activities. Several recent studies have shown the involvement of lncRNA in various human diseases, such as gastric cancer, prostate cancer, lung cancer, and so forth. However, obtaining lncRNA-disease relationship only through biological experiments not only costs manpower and material resources but also gains little. Therefore, developing effective computational models for predicting lncRNA-disease association relationship is extremely important. This study aimed to propose an lncRNA-disease association prediction model based on the weight matrix and projection score (LDAP-WMPS). The model used the relatively perfect lncRNA-miRNA relationship data and miRNA-disease relationship data to predict the lncRNA-disease relationship. The integrated lncRNA similarity matrix and the integrated disease similarity matrix were established by fusing various methods to calculate the similarity between lncRNA and disease. This study improved the existing weight algorithm, applied it to the lncRNA-miRNA-disease triple network, and thus proposed a new lncRNA-disease weight matrix calculation method. Combined with the improved projection algorithm, the lncRNA-miRNA relationship and miRNA-disease relationship were used to predict the lncRNA-disease relationship. The simulation results showed that under the Leave-One-Out-Cross-Validation framework, the area under the receiver operating characteristic curve of LDAP-WMPS could reach 0.8822, which was better than the latest result. Taking adenocarcinoma and colorectal cancer as examples, the LDAP-WMPS model was found to effectively infer the lncRNA-disease relationship. The simulation results showed good prediction performance of the LDAP-WMPS model, which was an important supplement to the research of lncRNA-disease association prediction without lncRNA-disease relationship data.

## Introduction

According to the traditional central principle, RNA is divided into messenger RNA (mRNA) and noncoding RNA (ncRNA). mRNA is the medium for DNA to be transcribed into a protein, while ncRNA has always been regarded as noise and has no real effect. However, the sequencing results showed that in the whole human gene pool, less than 5% of DNA and RNA were involved in protein transcription, while other genes were involved in RNA transcription that could not be encoded, that is, the number of ncRNA was far greater than that of coding RNA [1]. In 1998, two American scientists, Andrew Farr and Craig Mello, jointly published a paper on the discovery of RNA interference mechanism in the journal *Nature*. They believed that RNA interference existed in all organisms, and RNA played a regulatory role in gene expression [2], virus infection [3, 4], immune system [5], and so forth, thus bringing biological research into a new stage. Then, the research on ncRNA gradually increased, among which the research on long ncRNAs (lncRNAs) has been one of the hot topics. lncRNA is a kind of ncRNA whose nucleotide length is more than 200. In previous studies, it was considered to be the noise generated in the process of transcription [6, 7]. Nowadays, lncRNA has been found to be involved in all aspects of cell life cycle, including transcription [8], cell differentiation [9], cell transport [10], apoptosis [11], metabolic process [12], and so on. Moreover, lncRNA has also been found to be associated with various human diseases [13], including leukemia [14, 15], diabetes [16, 17], prostate cancer [18, 19], lung cancer [20, 21], colon cancer [22, 23], cardiovascular disease [24, 25], and so on. lncRNA participates in diseases through abnormal sequence and spatial structure, abnormal expression level, and abnormal interaction with binding proteins, thus affecting human health [26, 27].

Therefore, linking lncRNA with diseases can help realize the early detection of diseases, the targeted treatment of diseases, and the systematic understanding of the etiological characteristics of complex diseases. The biological experiments related to lncRNA cost a lot of money and time to carry out because of the complex lncRNA-disease relationship. Computer-aided experiment has become an effective research method. These experiments can effectively predict the complex lncRNA-disease relationship. The datasets in the open lncRNA database are used to verify the prediction results. The prediction of the lncRNA-disease relationship is of great significance in biology, medicine, and other fields. In the field of biology, computer-aided experiments can reduce the cost of experiments and improve the success rate of experiments. In the field of medicine, computer-aided experiments can help researchers identify lncRNAs related to various diseases and understand the pathogenesis of diseases at the molecular level so as to effectively prevent and treat diseases [28].

The proposed prediction model is developing rapidly. Many prediction models, including CircRNA-disease association prediction model [29], miRNA-disease association prediction model [30, 31], lncRNA-miRNA association prediction model [32, 33],and lncRNA-disease association prediction model. have greatly enriched the relationship between computer science and biology. This paper mainly proposes a new prediction method for lncRNA- disease association prediction. The following is an analysis of some previous lncRNA-disease association prediction models: The proposed model can be divided into two categories based on the experimental data. The first model relies only on the lncRNA-disease relationship information. Specifically, we can predict the lncRNA-disease relationship through the association information between lncRNAs and diseases. For example, Xie et al. proposed a new method for human lncRNA disease association prediction based on network consistent projection (NCPHLDA) [34]. The model integrates lncRNA cosine similarity network and disease cosine similarity network. At the same time, it has no requirements for parameters and has good prediction performance. However, there are some limitations. If the known lncRNA disease correlation is very small, the prediction results will be biased. Chen et al. developed the NCMCMDA [35] model, innovatively combines neighborhood constraints with matrix completion, providing a new idea to use similarity Information used to aid forecasting. However, NCMCMDA also has limitations. Currently known miRNA deficiency—disease association may affect the long road of NCMCMDA performance expansion data. Secondly, how to effectively select parameter information, miRNA similarity information and low order constraints to balance the influence of disease similarity still needs further research Based on disease semantic similarity and lncRNA-disease relationship information. Zhang et al. developed an LDAI-ISPS model to predict the potential lncRNA-disease relationship through network consistency [36]. The model integrated Gaussian interaction profile central similarity to calculate disease similarity and lncRNA similarity, which made up the incompleteness of the similarity network construction only with semantic similarity. However, this method still had limitations in that the predicted results were biased toward the diseases with more related lncRNAs or the lncRNAs with more related diseases. The other model integrated multiple data; collected multiple biological data, such as lncRNA, miRNA, protein, disease, and so on; and integrated these data into matrix or heterogeneous network to infer the potential lncRNA-disease relationship. For example, Fu et al. proposed an lncRNA disease association prediction method (MFLDA) based on matrix decomposition [37]. In this way, the weight of the data source and the correlation matrix of the disease can be assigned to the data source with smaller weight to speculate the potential association of lncRNA disease. The biggest advantage of this model is that it can easily predict the correlation between different research objects by classifying various heterogeneous data sources. However, MFLDA prefers to study data sparse matrix. Its performance depends on low-quality and irrelevant internal relational data sources, but it does not get rid of the use of lncRNA disease association attribute information. Yu and Wang et al. developed an NBCLDA model [38], which integrated a variety of organisms to construct a new tripartite network, including miRNA-disease, miRNA-lncRNA, and lncRNA-disease relationship and interaction. Then, a quadruple network was constructed, and a naïve Bayesian classifier was applied for the prediction. The important limitation of the naïve Bayesian classifier was that the information of negative samples was required. Therefore, unlabeled lncRNA-disease pairs were always randomly selected as negative samples, which could seriously influence the prediction performance. Yu et al. proposed a new model CFNBC [39], which was an improvement of the original NBCLDA model. It combined collaborative filtering with naïve Bayes and inferred the potential lncRNA-disease relationship by calculating the relationship score between lncRNA and disease. Although the introduction of a collaborative filtering algorithm effectively improved the prediction ability of CFNBC, it still failed to resolve the limitations of the naïve Bayesian model.

Most of the prediction of lncRNA-disease correlation needed to know the correlation between lncRNA and diseases. However, the known lncRNA-disease relationship is quite rare. To solve the aforementioned problems, this study proposed an lncRNA-disease association prediction model based on the weight matrix and projection score (LDAP-WMPS). The model used the relatively perfect lncRNA-miRNA relationship data and miRNA-disease relationship data to predict the lncRNA-disease relationship. The integrated lncRNA similarity matrix and the integrated disease similarity matrix were established by fusing various methods to calculate the similarity between lncRNA and disease. On this basis, the weight algorithm was improved and applied to the lncRNA-miRNA-disease triple network. Based on the network, a new lncRNA-disease weight matrix calculation method was proposed. Combined with the improved projection algorithm, the lncRNA-miRNA relationship and the miRNA-disease relationship were used to predict the lncRNA-disease relationship. The simulation results showed that based on the Leave-One-Out-Cross-Validation (LOOCV) framework, the area under the receiver operating characteristic (ROC) curve (AUC) of LDAP-WMPS could reach 0.8822, which was better than the latest result. Taking adenocarcinoma and colorectal cancer as examples, LDAP-WMPS was found to effectively infer the lncRNA-disease relationship.

## Materials and methods

### Dataset and preprocessing

The known lncRNA-disease relationship dataset was downloaded from the MNDRv2.0 database (2017 edition) [40]. The known miRNA-disease relationship datasets were downloaded from the HMDD database (2018 edition) [41]. The known lncRNA-miRNA relationship dataset was downloaded from the Starbase v2.0 database (2015 edition) [42]. After data cleaning and name unification, three datasets *D*_*LM*_, *D*_*MD*_, and *D*_*LD*_ were retrieved. The *D*_*LM*_ database comprised 1089 different lncRNAs and 246 different miRNAs; the *D*_*MD*_ database comprised 246 different miRNAs and 373 different diseases; and the *D*_*LD*_ comprised 1089 different lncRNAs and 373 different diseases. The *D*_*LD*_ dataset was not used as the training set, but only as the test set. The *D*_*MD*_ and *D*_*LM*_ datasets were analyzed and transformed into adjacency matrices. Taking lncRNA-miRNA relationship dataset as an example, the adjacency matrix A_*LM*_ was constructed. The lncRNA was listed as the row, and miRNA was listed as the column. If the miRNA in row j interacted with lncRNA in column i, then *A*_*LM*_(*i*,*j*) = 1; else, *A*_*LM*_(*i*,*j*) = 0. Similarly, the adjacency matrix *A*_*MD*_ was constructed.

### Cosine similarity for diseases

The principle of disease cosine similarity was based on the assumption that if disease *i* and disease *j* were similar to each other, which is a commonly used similarity calculation method [43].then the binary vectors *A*_*MD*_(:, *i*) and *A*_*MD*_(:, *j*) should also be similar to each other. The same assumption should also be true for diseases. According to the known miRNA-disease relationship data, the cosine similarity for disease between miRNA and disease was calculated as:

$$CD(i,j)=\frac{AMD(:,i)*A_{MD}(:,j)}{\left||A_{MD}(:,i)||||A_{MD}(:,j)|\right|}$$ (1)

Where *A*_*MD*_(:, *i*) is the *i*th column vector in the adjacency matrix of miRNA and disease, which represents the relationship feature of disease *i*.

### Jaccard similarity for diseases

Similarity measurement is the core of a prediction model. Cosine similarity is widely used in related prediction researches. However, in many practical applications, the sparsity of evaluation data is too high, and the calculation of cosine similarity between diseases produces misleading results. Compared with the traditional similarity measurement method, the Jaccard method improves the disadvantage that the cosine similarity only considers the disease score and ignores other information. It is especially suitable for data with high sparsity. The Jaccard similarity for disease between miRNA and disease was calculated as follows:

$$JD(i,j)=\frac{A_{\;MD}(:,i)\cap A_{\;MD}(:,j)}{A_{\;MD}(:,i)\cup A_{\;MD}(:,j)}$$ (2)

Where *A*_*MD*_(:, *i*) is the *i*th column vector in the adjacency matrix of miRNA and disease, which represents the relationship feature of disease i Similarly, *A*_*MD*_(:, *j*) represents the relationship feature of miRNA *j*; $A_{MD}(:,i)\cap A_{MD}(:,j)$is the number of miRNAs associated with disease *i* and disease *j*; and $A_{MD}(:,i)\cup A_{MD}(:,j)$ is the sum of miRNAs related to disease *i* and disease *j*.

### Integrated disease similarity

The two similarity calculation methods were integrated, the shortcomings of various similarity calculation methods were reduced to a certain extent, and the prediction ability of unknown relationships was greatly increased. Integrating disease semantic similarity and cosine similarity for diseases gave:

$$IDS(i,j)=\left\{\begin{array}{ll} CD(i,j) & if\;CD(i,j)\neq 0;\\ JD(i,j) & if\;CD(i,j)=0; \end{array}\right.$$ (3)

### Cosine similarity for lncRNA

Similar to the disease cosine similarity calculation method, the cosine similarity for lncRNA between lncRNA and miRNA was calculated as follows:

$$CL(i,j)=\frac{ALM(i,:)*A_{LM}(j,:)}{\left||A_{LM}(i,:)||||A_{LM}(j,:)|\right|}$$ (4)

Where *A*_LM_(*i*,:) is the *i*th row vector in the adjacency matrix of lncRNA and miRNA, which represents the relationship feature of lncRNA *i*.

### Jaccard similarity for lncRNA

Similar to the disease Jaccard similarity calculation method, the Jaccard similarity for lncRNA between lncRNA and miRNA was calculated as follows:

$$JL(i,j)=\frac{A_{LM}(i,:)\cap A_{LM}(j,:)}{A_{LM}(i,:)\cup A_{LM}(j,:)}$$ (5)

Where *A*_LM_(*i*,:) is the *i*th row vector in the adjacency matrix of lncRNA and miRNA, which represents the relationship feature of lncRNA *i*. Similarly, *A*_*LM*_(*j*,:) represents the relationship feature of lncRNA *j*; $A_{LM}(i,:)\cap A_{LM}(j,:)$ is the number of miRNAs associated with lncRNA *i* and lncRNA *j*; and $A_{LM}(i,:)\cup A_{LM}(j,:)$ is the sum of miRNAs related to lncRNA *i* and lncRNA *j*.

### Integrated lncRNA similarity

Similar to the disease-integrated similarity calculation method, integrating miRNA similarity MS and cosine similarity CL for lncRNA gave:

$$ILS(i,j)=\left\{\begin{array}{ll} CL(i,j) & if\;CL(i,j)\neq 0;\\ JL(i,j) & if\;CL(i,j)=0; \end{array}\right.$$ (6)

### Establishment of lncRNA-disease weight matrix

**Fig 1:**
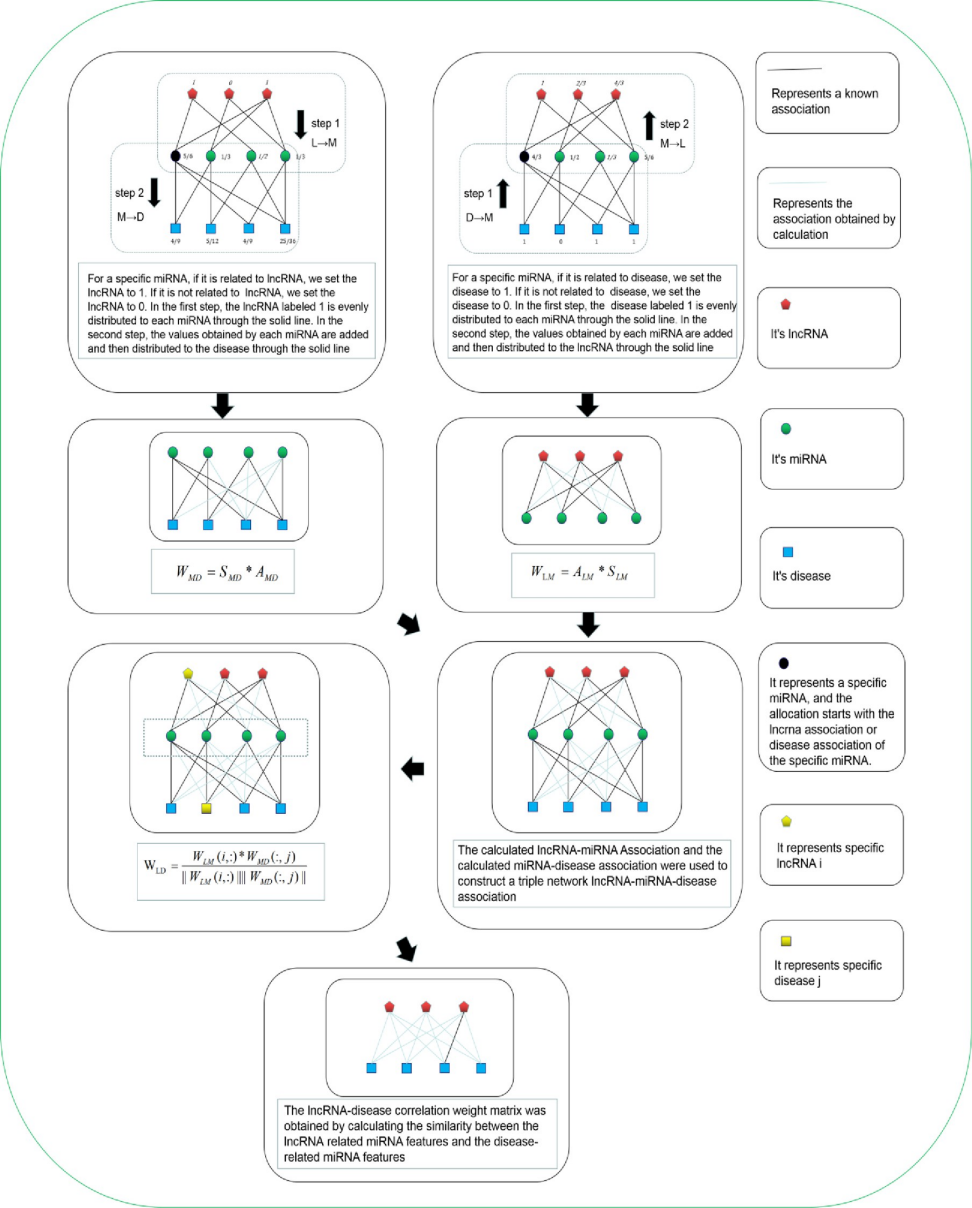
Flow chart of lnRNA-disease association weight matrix construction.

Weight assignment algorithm [44, 45] is often used in the association prediction of the lncRNA dual network. The correlation score between lncRNA and diseases could be obtained through weight distribution. This was further improved and applied to the lncRNA-miRNA-disease triple network, as shown in Fig 1. Taking L to M as an example, the first step was defined as follows:

$$f(M_{j})=\sum_{i=1}^{m}\frac{a_{ij}f(L_{i})}{k\;(L_{i})}$$ (7)

Where m is the number of lncRNAs, *k*(*L*_*i*_) gives the number of miRNAs related to lncRNA i, and *a*_*ij*_ represents an entity in the lncRNA-miRNA matrix *A*_*LM*_. *f*(*L*_*i*_) represents a binary vector formed by miRNA j corresponding to all lncRNA-miRNA relationships (if lncRNA is associated with miRNA, the value is 1; otherwise, the value is 0).

The second step was M to D, defined as:

$$f(D_{e})=\sum_{j=1}^{n}\frac{b_{je}}{k(M_{j})}\sum_{i=1}^{m}\frac{a_{ij}f(L_{i})}{k\;(L_{i})}$$ (8)

Where n is the number of miRNAs, and e is the number of diseases. *k*(*M*_*j*_) is the number of diseases related to miRNA j, and *b*_*je*_ represents an entity in the miRNA-disease matrix *A*_*MD*_.

*f*(*De*) could be expressed as:

$$f(D_{e})=\sum_{i=1}^{m}s^{md}*f(L_{i})$$ (9)

Combining Eqs (8) and (9), the following formula was obtained:

$$s^{md}=\frac{1}{k(L_{i})}\sum_{j=1}^{n}\frac{b_{je}a_{ij}}{k\;({\text{M}}_{j})}$$ (10)

In the aforementioned formula, *S*_*MD*_ = {*s*^*md*^}_n*n_ is the score of miRNA-disease relationship. The miRNA-disease relationship weight matrix was defined as:

$$W_{MD}=S_{MD}*A_{MD}$$ (11)

Similarly, the weight matrix *W*_*LM*_ from D to M to L was defined as:

$$s^{lm}=\frac{1}{k({\text{D}}_{\text{e}})}\sum_{j=1}^{n}\frac{b_{je}a_{ij}}{k\;(\text{M}j)}$$ (12)

$$W_{\text{L}M}=A_{LM}*S_{LM}$$ (13)

For lncRNA *i*, the potential relationship characteristics between miRNAs and lncRNA *i* were calculated; and for disease *j*, the potential relationship characteristics between miRNAs and disease *j* were also calculated. *W*_L*M*_(*i*,:) was used to represent the eigenvalue of miRNA associated with lncRNA *i*, and *W*_*MD*_(:, *j*) to represent the eigenvalue of miRNA associated with disease *j*. Then, the weight between lncRNA and disease was defined as follows:

$$w^{ij}=\frac{W_{LM}(i,:)*W_{MD}(:,j)}{\left||W_{LM}(i,:)||||W_{MD}(:,j)|\right|}$$ (14)

### Building LDAP-WMPS prediction model

**Fig 2:**
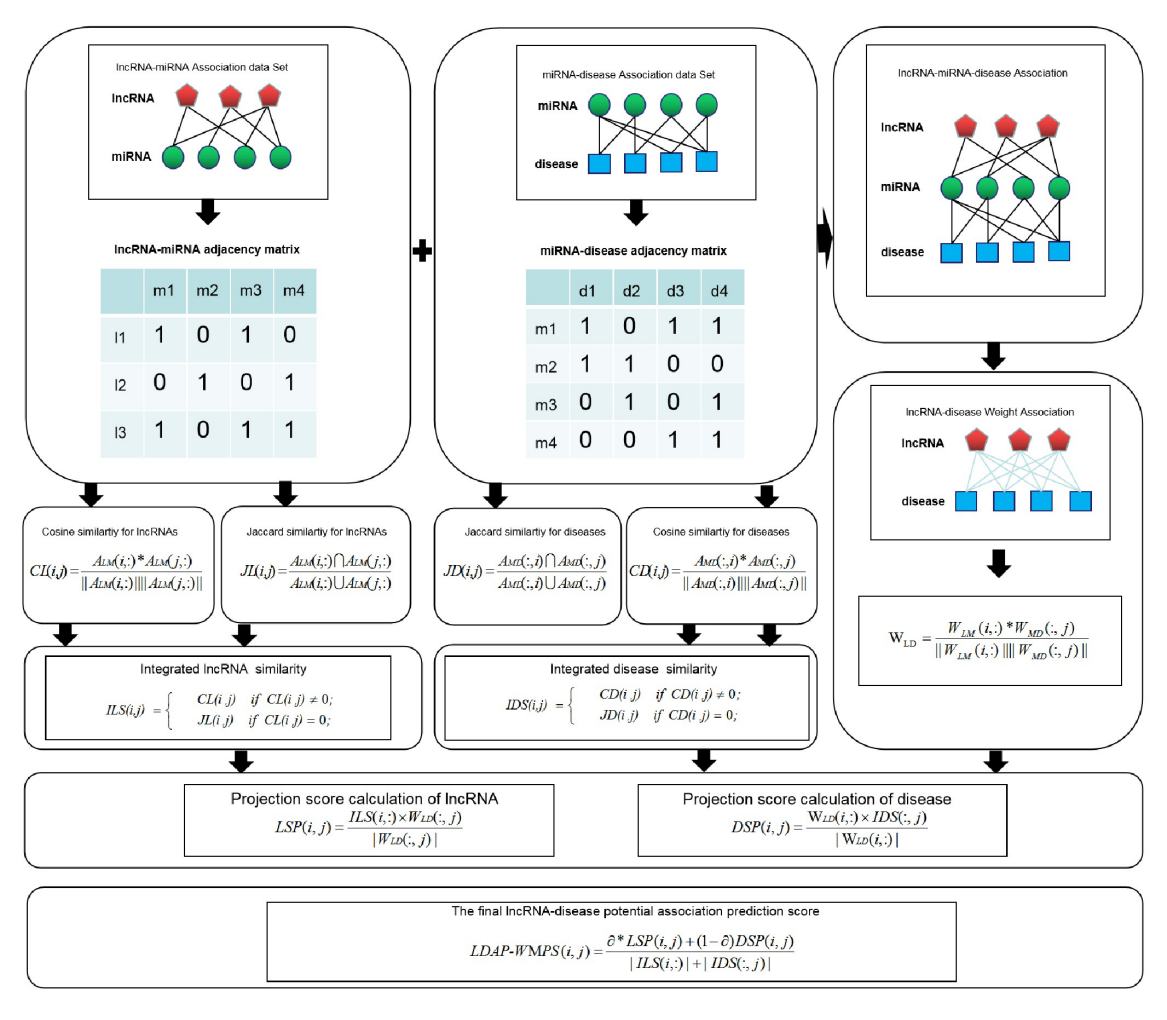
Flow chart of LDAP-WMPS applied to lncRNA-disease association prediction.

The flowchart of the LDAP-WMPS model is shown in Fig 2. The LDAP-WMPS model was divided into three parts: the first step calculated the disease projection score; the second step calculated the lncRNA projection score; and the third step fused the disease projection score and the lncRNA projection score proportionally, which were then normalized to get the prediction score matrix [46].

The disease projection score was defined by the following formula:

$$DSP(i,j)=\frac{W_{LD}(i,:)\times IDS(:,j)}{\left|W_{LD}(i,:)\right|}$$ (15)

where *W*_*LD*_(*i*,:) is the vector formed by the *i*th row of the lncRNA-disease weight matrix, which represents the relationship score between lncRNA *i* and various diseases. Its calculation process is shown in Fig 1. *IDS*(:, *j*) is the vector formed by column *j* of the integrated disease similarity matrix, which represents the vector composed of the similarity between disease *j* and other diseases. |W_*LD*_(*i*,:)| represents the module length of disease *i*-related lncRNA component vector. *DSP*(*i*, *j*) is the projection score of the disease. The multidimensional similarity relation was transformed into a concrete value by projection.

The projection score of lncRNA was defined as follows:

$$LSP(i,j)=\frac{ILS(i,:)\times W_{LD}(:,j)}{\left|W_{LD}(:,j)\right|}$$ (16)

In the aforementioned formula, *ILS*(*i*,:) is the vector formed by the *i*th row of the functional similarity matrix of lncRNA, which represents the vector composed of the similarity between lncRNA *i* and other kinds of lncRNA. W_LD_(:, *j*) is the vector formed by column *j* of lncRNA-disease relationship weight matrix, which represents the relationship score between disease *j* and various lncRNAs. |W_LD_(:, *j*)| is the module length of lncRNA *i*-related disease component vector. *LSP*(*i*, *j*) is the projection score of lncRNA.

The final lncRNA-disease potential association prediction score matrix was formed by fusing lncRNA projection score with the disease projection score, defined as:

$$LDAP-WDPS(i,j)=\frac{\partial *LSP(i,j)+(1-\partial )DSP(i,j)}{\left|ILS(i,:)|+|IDS(:,j)\right|}$$ (17)

where *LDAP*-*WDPS*(*i*, *j*) is the final relationship score between lncRNA *i* and disease *j*. |ILS(*i*,:)| is the module length of the lncRNA composition vector similar to lncRNA *i* in integrated lncRNA similarity matrix, and |IDS(:, *j*)| is the module length of the disease composition vector similar to disease *j* in integrated disease similarity matrix. ∂ is the proportion of the lncRNA projection score and the disease projection score in the fusion score calculation.

## Results

### Performance evaluation

The performance of the LDAP-WMPS model was evaluated using the LOOCV framework, and each known disease-lncRNA relationship was left out in turn as a test sample. How well this test sample was ranked relative to the candidate samples (all the disease-lncRNA pairs without the evidence to confirm their relationships) was evaluated. When the rank of this test sample exceeded the given threshold, this model was considered to provide a successful prediction. The evaluation process (LOOCV) came from the reference [39]. For a more detailed code, the code was link in reference [39]. The results were compared with other prediction models using LOOCV, and with other prediction models for LOOCV. The true-positive rate (TPR) and false-positive rate (FPR) were calculated to obtain the ROC and the AUC for intuitive evaluation:

$$TPR=\frac{TP}{TP+FN}$$ (18)

$$FPR=\frac{FP}{FP+TN}$$ (19)

The ROC curve was drawn with TPR and FPR, and the AUC was calculated.

### Comparison with other advanced models

**Fig 3:**
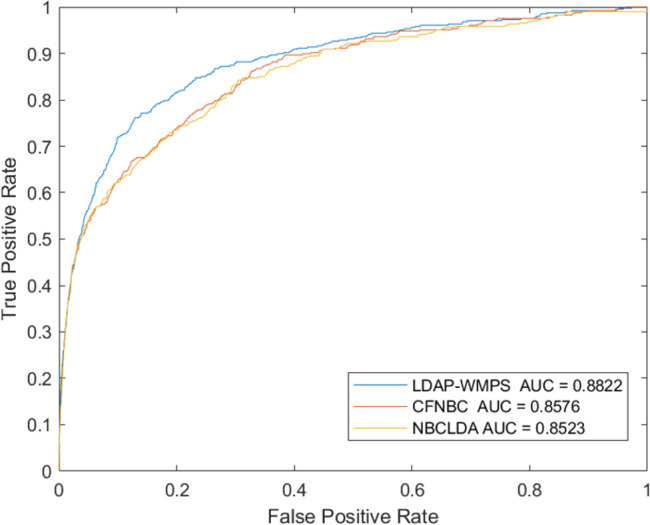
The performance of LDAP-WMPS and others models in terms of ROC curves and AUCs based on 407 known lncRNA-disease associations under the framework of LOOCV.

**Fig 4:**
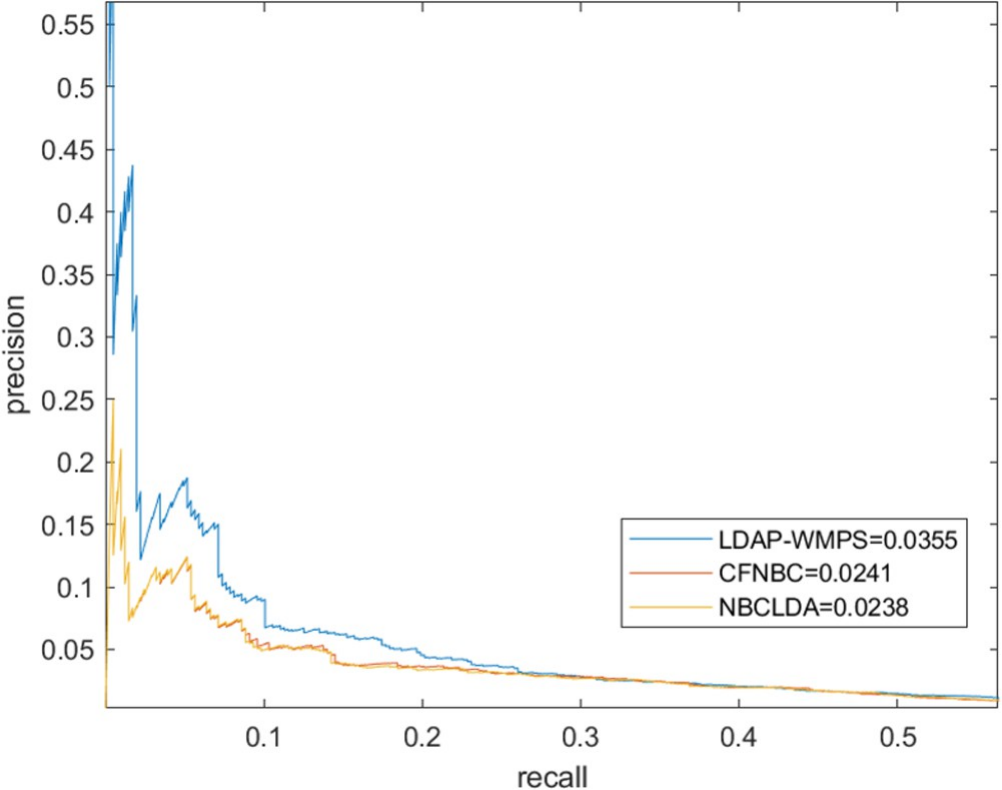
The performance of LDAP-WMPS and others models in terms of PR curves and AUPRs based on 407 known lncRNA-disease associations under the framework of LOOCV.

The LDAP-WMPS model was compared with other advanced models to prove the effectiveness of the LDAP-WMPS model. Considering that the dataset used in this model was the same as those of NBCLDA [38] and CFNBC [39] models, the NBCLDA and CFNBC models were chosen as the comparison models. The ROC and AUC were obtained by applying three different models to the same dataset. After comparison, the LDAP-WMPS model was slightly better than the other methods in the ROC curve, and the AUC reached the value 0.8822. The highest AUC values of the NBCLDA and CFNBC models were 0.8521 and 0.8576, respectively. The results showed that the proposed method was slightly better than the CFNBC method. The results are shown in Table 1 and Figs 3 and 4. All models were statistically tested, and the P value was less than 0.05, indicating that all models had statistical significance.

**Table 1:** AUC values of LDAP-WMPS model and other models under LOOCV framework under the same dataset.

Method	AUC	AUPR	P value
LDAP-WMPS	0.8822	0.0355	<0.05
NBCLDA	0.8521	0.0238	<0.05
CFNBC	0.8576	0.0241	<0.05

### Analysis of parameters

**Fig 5:**
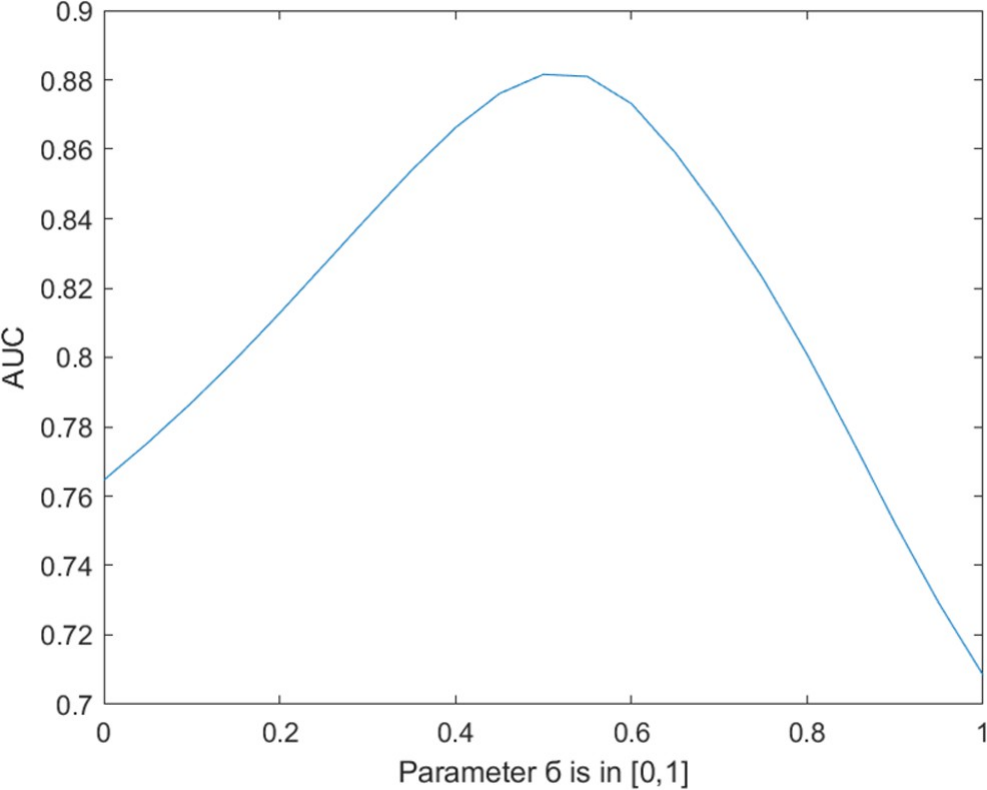
Influence curve of parameter a on AUC within [0,1].

**Fig 6:**
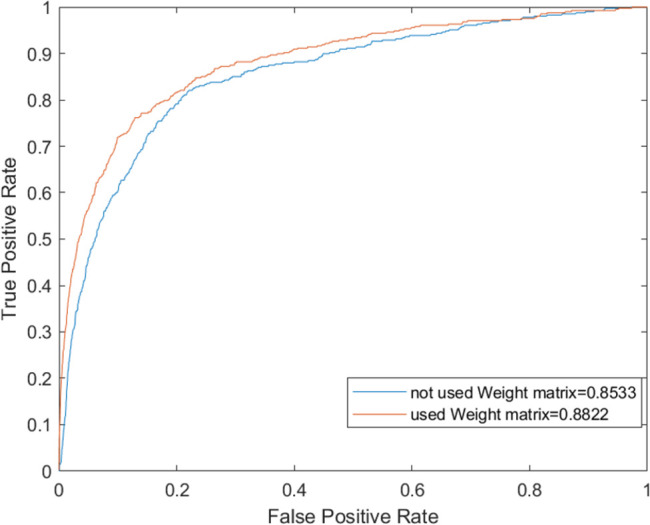
Comparison of ROC curve calculated with weight matrix and ROC curve calculated without weight matrix.

In the proposed model, a parameter ∂ was introduced. The range of the parameter ∂ was [0,1]. When ∂ = 0, only the disease projection score was used for the final score calculation; when ∂ = 1, only the lncRNA projection score was used for the final score calculation. The results are shown in Fig 5. Obviously, when ∂ = 0.52, AUC reached the highest value of 0.8822. The models using and not using weight matrix were evaluated, respectively, to further prove the effectiveness of the proposed lncRNA-disease weight matrix, and the results are shown in Fig 6. It was obvious that the weight matrix of the proposed model effectively improved the prediction ability.

### Case studies

Tumor refers to a new organism formed by the proliferation of local tissue cells under the action of various oncogenic factors because this new organism is mostly space-occupying massive protuberance, also known as vegetation. According to the cellular characteristics of tumors and the degree of harm to the body, tumors are divided into benign tumors and malignant tumors: benign tumors can be removed by surgery and do not metastasize and relapse. Malignant tumors, often called cancer, are easy to metastasize, difficult to cure by surgery, and have a possibility of recurrence after cure [47]. Adenocarcinoma and colorectal cancer were studied to further prove the practicability of LDAP-WMPS in lncRNA-disease association prediction. The first 20 pieces of information about LDAP-WMPS predicting adenocarcinoma and colorectal cancer are shown in Tables 2 and 3, respectively.

**Table 2:** Top 20 lncRNA of colorectal neoplasms predicted by LDAP-WMPS.

lncRNA	Evidence (PMID)	Rank
XIST	28837144	1
MALAT1	25031737;21503572	3
DCP1A	29964337	4
KCNQ1OT1	16965397;11340379	5
NEAT1	30185232	8
OIP5-AS1	29773344	9
HCG18	31854468	10
FGD5-AS1	31332696	13
TUG1	31528224	14
RP4-773N10.5	31966592	15
SNHG16	32859986	18
GAS5	31619268	20

**Table 3:** Top 20 lncRNA of adenocarcinoma predicted by LDAP-WMPS.

lncRNA	Evidence (PMID)	Rank
XIST	28961027	1
MALAT1	31480991	3
DCP1A	25089265	4
RP6-24A23.7	28299977	5
KCNQ1OT1	30932685	7
HCG18	32559619	8
NEAT1	30036873	9
OIP5-AS1	32669972	10
CTB-89H12.4	26975529	12
FGD5-AS1	33416094	13
SNHG16	31580045	16
SENP3-EIF4A1	32602848	17
TUG1	29960845	18
LINC00662	33108738	20

Colorectal cancer is a common cancer type. Its incidence rate and mortality rate are high in the world. In 2018 alone, the number of new cases reached nearly 2 million, and the number of deaths was nearly 900,000. Some data showed that about 5.2% of men and 4.8% of women were at risk of colorectal cancer in the United States, and the mortality caused by colorectal cancer was close to 33% [48]. Many studies showed that lncRNA was closely related to colorectal cancer. In the prediction results of this study, 12 of the first 20 lncRNAs associated with colorectal cancer had been already proved by relevant medicine: lncRNA XIST expedited metastasis and modulated epithelial-mesenchymal transition in colorectal cancer [49]; lncRNA SNHG16 promoted colorectal cancer cell proliferation, migration, and epithelial-mesenchymal transition through miR-124-3p/MCP-1 [50]; and lncRNA MALAT1 promoted the colorectal cancer malignancy by increasing DCP1A expression and miR203 downregulation [51]. The lncRNA HCG18 promoted the growth and invasion of colorectal cancer cells through sponging miR-1271 and upregulating MTDH [52]. lncRNA FGD5-AS1 promoted colorectal cancer cell proliferation, migration, and invasion through upregulating CDCA7 via sponging miR-302e [53]. lncRNA TUG1 mediated 5-fluorouracil resistance by acting as a competing endogenous RNA of miR-197-3p in colorectal cancer [54].

Adenocarcinoma is a kind of lung cancer. It is least related to smoking, accounting for 40% of primary adenocarcinoma. It is often located in the peripheral part of the lung, but also involves the pleura and the formation of associated scarring and pleural effusion. Extensive resection should be performed because of the invasive growth of adenocarcinoma. The rate of lymph node metastasis of adenocarcinoma is high, which can be as high as 36%-47%. It is easy to relapse and has a poor prognosis. Lin Guoji reported 68 cases of adenocarcinoma. The 5-year and 10-year cure rates were 43.9% and 29.0%, respectively [55]. In the prediction results of the proposed model, 14 of the first 20 lncRNAs associated with adenocarcinoma had been already proved by relevant medicine: lncRNA XIST promoted human lung adenocarcinoma cells to cisplatin resistance via let-7i/BAG-1 axis [56]; lncRNA MALAT1 promoted gastric adenocarcinoma through the miR-181a-5p/AKT3 axis [57]; lncRNA CTB-89H12.4 regulated phosphatase and tensin homolog expression in prostate cancer [58]; lncRNA HCG18 acted on the oncogene in lung adenocarcinoma and enhanced lung adenocarcinoma progression by targeting miR-34a-5p/HMMR axis [59]; and lncRNA SNHG16 promoted cell proliferation and invasion in lung adenocarcinoma via sponging let-7a-5p [60].

**Fig 7:**
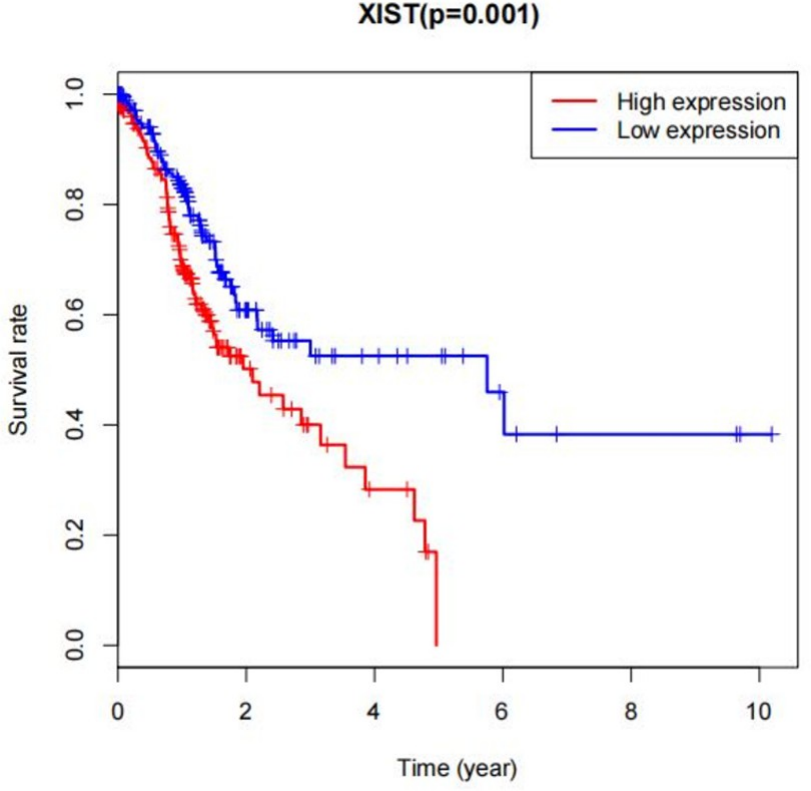
Survival period of XIST in high and low expression.

Next, we took the XIST gene as an example for further analysis to verify whether it might be associated with Colorectal cancer. In our study, we divided all Colorectal cancer patient samples into high and low expression groups. Tis phenomenon was observed by survival analysis. Tat, the survival time of Colorectal cancer patients in the XIST gene high expression group was relatively short, as shown in Fig 7.

## Discussion

Investigating the lncRNA-disease relationship is not only of great significance to the treatment of diseases but also helpful to explore the mystery of the human body. Using artificial intelligence to mine the existing medical data not only improves the use rate of data but also speeds up the process of medical intelligence. In this study, a computational model LDAP-WMPS was proposed. In this model, a weight allocation algorithm based on the lncRNA-miRNA-disease triple network and an lncRNA-disease relationship weight calculation method were proposed. The lncRNA-disease weight matrix was combined with the improved projection algorithm to calculate the relationship between each lncRNA and disease the interaction between lncRNA and disease information was obtained. Compared with the other three models, LDAP-WMPS was slightly better in AUC. Twelve of the first 20 lncRNAs were confirmed to predict the relationship between adenocarcinoma and colorectal cancer, which also proved the reliability of LDAP-WMPS. In addition, the proposed model was based on the lncRNA-miRNA relationship and miRNA-disease relationship to achieve the prediction of the lncRNA-disease relationship. The present relatively perfect lncRNA-miRNA relationship dataset and miRNA-disease relationship dataset to predict the lncRNA-disease relationship could effectively avoid the current lack of lncRNA-disease relationship data in data prediction. However, the proposed model also had some limitations. Many kinds of data were required for prediction, such as the miRNA-disease relationship dataset and lncRNA-disease relationship dataset. At the same time, the density of the aforementioned two datasets had a great impact on the final prediction results.

## Conclusions

The main contributions of this study were as follows: (1) An integrated lncRNA similarity calculation method and an integrated disease similarity calculation method were proposed. The similarity was calculated by a variety of similarity calculation methods, which could effectively avoid the defects of insufficient similarity obtained by a single similarity calculation method and improve the prediction ability of the model to unknown relationships. (2) Based on the weight distribution of lncRNA-miRNA-disease triple network, a method of lncRNA-disease weight calculation was proposed. This method could effectively associate lncRNA-miRNA dataset with miRNA-disease dataset and help in indirectly predicting lncRNA-disease relationship through lncRNA-miRNA dataset and miRNA-disease dataset. (3) The existing consistency projection scoring formula was improved, and the proportion of the projection of the lncRNA part and the projection of disease part was adjusted in the final score to improve the prediction ability. (4) The lncRNA-disease relationship could be predicted by the LDAP-WMPS model without relying on the known lncRNA-disease relationship data.

## Supporting information

S1 Data (ZIP)

## Abbreviations

AUC: areas under ROC curve
LDAP-WMPS: lncRNA-Disease Association Prediction Based On Weight Distribution And Projection Score
PR: false positive rates
LMDN: the lncRNA-miRNA-disease tripartite network
LMDN: an updated lncRNA-miRNA-disease association tripartite network
lncRNA: long non-coding RNAs lncRNA
LOOCV: Leave-One Out Cross Validation
PR: true positive rates
